# Physical interface dynamics alter how robotic exosuits augment human movement: implications for optimizing wearable assistive devices

**DOI:** 10.1186/s12984-017-0247-9

**Published:** 2017-05-18

**Authors:** Matthew B. Yandell, Brendan T. Quinlivan, Dmitry Popov, Conor Walsh, Karl E. Zelik

**Affiliations:** 1 Department of Mechanical Engineering, Vanderbilt University, 2301 Vanderbilt Place, PMB 401592, Nashville, TN 37240-1592 USA; 2 Harvard University, Harvard John A. Paulson School of Engineering and Applied Sciences, Pierce Hall, 29 Oxford Street, Cambridge, MA 02138 USA; 3 Department of Biomedical Engineering, Vanderbilt University, 2301 Vanderbilt Place, PMB 401592, Nashville, TN 37240-1592 USA; 4 Department of Physical Medicine and Rehabilitation, Vanderbilt University, 2301 Vanderbilt Place, PMB 401592, Nashville, TN 37240-1592 USA

**Keywords:** Exoskeleton, Wearable robot, Rehabilitation, Power transfer, Soft tissue, Joint kinetics, Human augmentation, Physical human-robot interaction

## Abstract

**Background:**

Wearable assistive devices have demonstrated the potential to improve mobility outcomes for individuals with disabilities, and to augment healthy human performance; however, these benefits depend on how effectively power is transmitted from the device to the human user. Quantifying and understanding this power transmission is challenging due to complex human-device interface dynamics that occur as biological tissues and physical interface materials deform and displace under load, absorbing and returning power.

**Methods:**

Here we introduce a new methodology for quickly estimating interface power dynamics during movement tasks using common motion capture and force measurements, and then apply this method to quantify how a soft robotic ankle exosuit interacts with and transfers power to the human body during walking. We partition exosuit end-effector power (i.e., power output from the device) into power that augments ankle plantarflexion (termed augmentation power) vs. power that goes into deformation and motion of interface materials and underlying soft tissues (termed interface power).

**Results:**

We provide empirical evidence of how human-exosuit interfaces absorb and return energy, reshaping exosuit-to-human power flow and resulting in three key consequences: (i) During exosuit loading (as applied forces increased), about 55% of exosuit end-effector power was absorbed into the interfaces. (ii) However, during subsequent exosuit unloading (as applied forces decreased) most of the absorbed interface power was returned viscoelastically. Consequently, the majority (about 75%) of exosuit end-effector work over each stride contributed to augmenting ankle plantarflexion. (iii) Ankle augmentation power (and work) was delayed relative to exosuit end-effector power, due to these interface energy absorption and return dynamics.

**Conclusions:**

Our findings elucidate the complexities of human-exosuit interface dynamics during transmission of power from assistive devices to the human body, and provide insight into improving the design and control of wearable robots. We conclude that in order to optimize the performance of wearable assistive devices it is important, throughout design and evaluation phases, to account for human-device interface dynamics that affect power transmission and thus human augmentation benefits.

**Electronic supplementary material:**

The online version of this article (doi:10.1186/s12984-017-0247-9) contains supplementary material, which is available to authorized users.

## Background

Soft exosuits and rigid exoskeletons, two types of wearable assistive devices, have demonstrated the potential to improve mobility outcomes for individuals with disabilities [1–3], and to augment healthy human performance [3–6]. Human performance might be further enhanced by increasing the mechanical power provided by these wearable technologies [3, 6–8] in conjunction with appropriate control strategies [7, 8]. However, the power generated by wearable assistive devices will only enhance human performance if it is effectively transferred from the device to the user via a physical interface. Growing evidence indicates that inefficient device-to-human power transmission is a critical problem for wearable assistive devices [7, 9], which undermines potential health and performance benefits.

Transmitting power from an assistive device to the human body is challenging because biological tissues and interfaces deform and displace when forces are applied, absorbing power. For soft exosuits, coupling to the human body can be challenging because it often involves affixing physical interfaces (e.g., straps, sleeves) around body segments and transmitting loads partially in shear, which stretches the underlying soft tissues. The physical interface itself (often constructed of soft or compliant materials) can also stretch and migrate (i.e., slip) relative to the skin [10–12]. Collectively, the biological tissues and interface materials deform and move relative to each other, resulting in power absorption (and return) which we refer to here as *interface power*. Rigid exoskeletons face an analogous challenge, though forces are often oriented more orthogonally to human body segments. Body segments in this context tend to absorb power via compression of soft tissues [13], but may also experience shear loading and deformation due to factors such as misalignment between human and exoskeleton joint centers [3, 11–14]. Designing devices that load bony landmarks or orient forces so as to minimize soft tissue deformation (e.g., [11, 15]) can help to reduce interface power absorption; however, this is not always possible. Landmarks are not always conveniently located (e.g., when attaching to the thigh), and in some cases high loads on small bony prominences can lead to discomfort [10, 11]. Thus, physical interfaces and associated transmission power losses represent a ubiquitous challenge for wearable assistive devices.

Inefficient device-to-human power transmission substantially undermines the performance benefits of wearable assistive devices. Experiments on a recent running exoskeleton found that about 50% of the mechanical power provided by the device was lost in transmission to the body [7, 9]. Thus a large portion of exoskeletal power was not used to augment running, but rather was absorbed in soft tissues, interface dynamics, and motion of the device relative to the user’s body. The authors identified the physical interface as one of the key factors that led to decreased exoskeleton performance, and ultimately concluded that this interface is one of the “major roadblocks to designing successful lower limb robotic exoskeletons” [9]. It remains critical to develop an improved understanding of these interface dynamics, which will have important implications for how wearable devices are physically coupled to the body and how they are controlled to effectively transfer power to assist human movement.

Although the practical difficulties of physically coupling wearable devices to the human body are well-known, only a few studies have published objective data characterizing interface dynamics [11, 16, 17], due partly to the lack of methods to quickly estimate these quantities. Human-device interface dynamics are not captured via standard gait analysis techniques or with conventional motion capture marker sets. For instance, standard rigid-body inverse dynamics approaches can estimate net power at the joints, but do not estimate power flow between the human body and the device acting in parallel. We have previously estimated interface power (between exosuit and human) using a series of quasi-static experiments to isolate absorption from each individual system component, both synthetic and biological. This was accomplished by sequentially characterizing: (a) raw textile material dynamics using tensile testing, (b) Bowden cable dynamics using an instrumented actuator unit, (c) exosuit interface dynamics on a mannequin, and finally (d) interface dynamics while the device was being worn by a human subject (thus capturing contributions from both the exosuit interface textiles and underlying biological tissues) [17]. These data were used to estimate stiffness and damping parameters for each component, which were then used to model contributions during dynamic movement [11, 17]. Building upon this previous work, it would be beneficial to develop new analysis methods that quantify device-to-human power transmission dynamics without needing to perform a series of time-consuming, component-by-component pre-experiments. The objective of this paper is two-fold: first to present a new methodology for quickly estimating interface power during dynamic tasks using common motion capture and force measurements, and second to apply this method to quantify how a soft robotic exosuit interacts with and transfers power to the human body during locomotion.

## Methods

We performed a motion analysis study using a previously developed soft robotic exosuit [18], then employed a new biomechanical analysis to evaluate exosuit-to-human power transmission, which enabled us to parse augmentation power (powering ankle plantarflexion) vs. interface power (absorbed into deformation and/or motion of the exosuit interface materials and underlying human soft tissues) during walking.

### Exosuit hardware

**Fig. 1:**
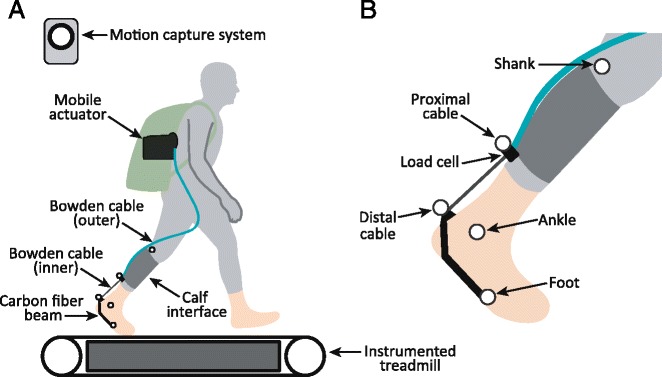
Experimental setup. **a** Human subject walked on a force-instrumented treadmill while wearing a robotic exosuit that assists ankle plantarflexion. **b** Simplified representation of motion capture markers used for power calculations. For graphical simplicity, a single marker is used to represent the shank, and a single marker is used to represent the foot; however, in practice segmental kinematics were estimated from several markers distributed along each segment (as detailed in Methods text and depicted in Additional file 1). These markers were selected to mitigate confounds due to soft tissue motion

The robotic exosuit has been fully detailed in previous work [4, 11, 17] so we only briefly summarize the hardware here. The exosuit was configured to provide unilateral ankle plantarflexion assistance (Fig. 1). The exosuit used in this study consists of 4 major components: (i) a mobile actuation unit (actuator plus controller) mounted to a backpack, (ii) a Bowden cable that transmits actuator power down to the ankle, (iii) an interface that attaches the outer sheath of the Bowden cable to the user’s shank, and (iv) a modified boot that connects the inner Bowden cable to the user’s foot via a cantilevered carbon fiber beam, such that the cable can provide a plantarflexion torque about the ankle. The system was instrumented with a load cell (LTH300, FUTEK Advanced Sensor Technology, Irvine, CA, USA) to measure forces in the Bowden cable at the end-effector (Fig. 1), and an encoder to measure Bowden cable displacement at the actuation unit. Two gyroscope sensors (LY3100ALH, STMicroelectronics, Geneva, Switzerland) were also affixed to the boot, and used to provide real-time input to the exosuit controller.

### Data collection & processing

The methods were evaluated on one healthy male subject (age: 27 years old, mass: 74 kg, height: 1.8 m) during walking, while collecting synchronous motion capture, motor encoder, load cell, and ground reaction force data (Fig. 1). The study was approved by the Harvard Longwood Medical Area Institutional Review Board, and all methods were carried out in accordance with the approved study protocol. The subject provided written informed consent before his participation and after the nature and possible consequences of the studies were explained. Kinematics were measured using a reflective marker motion capture system (Vicon, Oxford Metrics, Oxford, UK; 120 Hz). Three-dimensional ground reaction forces were measured using an instrumented split-belt treadmill (Bertec, Columbus, OH, USA; 2160 Hz). All marker and ground reaction force data were filtered using zero-lag 4th order low pass Butterworth filters with 8 Hz cut-off frequencies. Exosuit cable forces were recorded at 60 Hz via load cell. 3D ankle angles, moments and power were calculated for the right leg using conventional kinematic and inverse dynamics analyses (Visual3D, C-Motion, Rockville, MD, USA) [19, 20].

### Walking trial & exosuit controller

The subject walked on the treadmill at 1.5 m/s for 5 min using a previously published walking controller to apply peak cable force of up to 500 N that with an 8.4 cm moment arm equated to an approximate joint torque of 42 Nm [18]. In brief, the controller used real-time load cell, motor encoder, and gyroscope data to perform force-based position control of the cable on a step-by-step basis [18]. Cable pre-tensioning occurred during early to mid-stance, then the exosuit generated impulsive ankle plantarflexion torque and power during the push-off (end of stance) phase of gait [21]. At the very beginning of the trial no cable forces were applied. Over the course of about the first minute the peak cable force during push-off gradually increased from 0 to the desired force of 500 N, then for the remaining 4 min peak forces of 500 N were applied. For this subject and gait speed, the forces were applied at ~1 Hz (stride frequency).

### Analysis overview

Here we present a new methodology that enables us to estimate and understand exosuit-to-human power transmission during movement using common gait analysis measures (e.g., kinematics, forces). First, we discuss an estimation of the power generated by the robotic exosuit end-effector (i.e. the power output at the distal end of the Bowden cable). Second, we present an estimation of interface power, the power absorbed into the proximal (shank) and distal (foot) interfaces. Power absorbed into these interfaces is due to deformation of interface materials and underlying biological tissues, and relative motion of the interface with respect to the body. Therefore, when we discuss interface power we are referring to the combined behavior of synthetic materials and biological tissues. Third, we detail estimation of augmentation power, which contributes to augmenting joint mechanics (in this case, ankle plantarflexion). Fourth, we combine augmentation power with conventional inverse dynamics to estimate biological vs. exosuit contributions to net ankle power during gait. Fifth, for comparison purposes we define idealized augmentation power, which assumes no interface losses. Finally, we detail key summary metrics.

### Cable end-effector power

Cable end-effector power, *P*_*cable*_*end*_, was computed as the dot product of the cable force vector, ${\overset{\rightharpoonup }{F}}_{cable}$, and the cable velocity vector, ${\overset{\rightharpoonup }{v}}_{cable\_ end}$ (see Additional file 1 for comprehensive definitions and derivations).

1 $$ {P}_{cable\_ end}={\overset{\rightharpoonup }{F}}_{cable}\cdot {\overset{\rightharpoonup }{v}}_{cable\_ end} $$

**Fig. 2:**
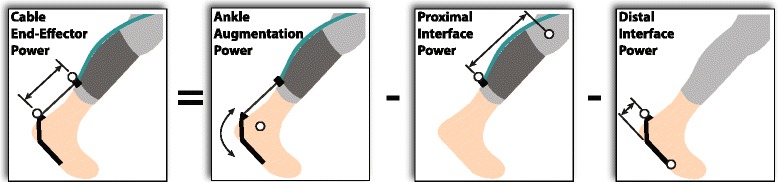
Conceptual summary of exosuit-to-human power transmission. Power is generated at the cable end-effector. A portion of this power contributes to ankle plantarflexion (termed ankle augmentation power), while a portion is absorbed into the human-exosuit interfaces (termed proximal and distal interface powers). Power absorbed into the proximal (shank) and distal (foot) interfaces is due to viscoelastic deformation of interface materials and underlying biological tissues, as well as relative motion of the interface with respect to the body. Reporting convention: power absorbed by the interfaces is negative, and power returned by the interfaces is positive. Black arrows represent motions associated with each power term

Cable end-effector power can contribute to augmentation power (in this study, augmenting ankle joint rotation), or alternatively it can be absorbed via (proximal or distal) interface power (Fig. 2).

### Proximal (shank) interface power

To estimate the cable end-effector power that is absorbed into (and then returned by) the proximal interface we can apply the classical definition of mechanical power (and work), by considering the velocity (and displacement) of the point of force application in the direction of said force. In the simplest case, in which the shank is fixed in 3D space, the proximal interface power could simply be estimated as the dot product of the cable force, ${\overset{\rightharpoonup }{F}}_{cable}$ (see Additional file 1 for calculation details), and the absolute velocity of the proximal cable marker, ${\overset{\rightharpoonup }{v}}_{prox\_ cable}$, since this is where the force is applied to the proximal interface (Fig. 1b). However, if the leg is moving in space (e.g., during walking), then it is necessary to quantify the velocity of the force application point relative to the shank’s gross (rigid-body) motion, which we term the *proximal interface velocity*, ${\overset{\rightharpoonup }{v}}_{prox\_ int}$. This captures the velocity changes due to non-rigid deformation and relative motion of the interface and soft tissues, while excluding the velocity changes due to shank segment translation and rotation. For this generalized case, ${\overset{\rightharpoonup }{v}}_{prox\_ int}$ simplifies to the following expression:

2 $$ {\overset{\rightharpoonup }{v}}_{prox\_ int}=\left({\overset{\rightharpoonup }{v}}_{shank}-{\overset{\rightharpoonup }{v}}_{prox\_ cable}\right) - {\overset{\rightharpoonup }{\omega}}_{shank} \times \left({\overset{\rightharpoonup }{\rho}}_{shank}-{\overset{\rightharpoonup }{\rho}}_{prox\_ cable}\right) $$

The absolute translational velocity of the proximal cable marker is represented by ${\overset{\rightharpoonup }{v}}_{prox\_ cable}$, while ${\overset{\rightharpoonup }{v}}_{shank}$ and ${\overset{\rightharpoonup }{\omega}}_{shank}$ are the absolute translational and rotational velocities of the shank segment, respectively. Finally, ${\overset{\rightharpoonup }{\rho}}_{prox\_ cable}$ and ${\overset{\rightharpoonup }{\rho}}_{shank}$ are the absolute positions of the proximal cable marker and shank in the lab coordinate frame, respectively. Experimentally, shank kinematics were estimated assuming rigid-body motion and using six motion capture markers that were placed on the skin (two located on the femoral epicondyles of the knee, two on the ankle malleoli and two along the shank segment). Shank markers were placed to avoid confounds due to localized tissue/skin stretch resulting from interface loading (see Additional file 1: Figure S1).

Finally, the *proximal interface power* was computed as the dot product of the cable force vector, ${\overset{\rightharpoonup }{F}}_{cable}$, and this proximal interface velocity, ${\overset{\rightharpoonup }{v}}_{prox\_ int}$.

3 $$ {P}_{prox\_ int}={\overset{\rightharpoonup }{F}}_{cable}\cdot {\overset{\rightharpoonup }{v}}_{prox\_ int} $$

We used a convention whereby power absorbed by the interface was negative, and power returned by the interface was positive.

### Distal (foot) interface power

Analogous calculations can be performed to estimate *distal interface velocity*, ${\overset{\rightharpoonup }{v}}_{dist\_ int}$, and *distal interface power*, *P*_*dist*_*int*_, due to deformation of the boot and attached cantilevered beam. Four markers on the foot segment were used to track segmental kinematics (two on the ankle malleoli, one on the first metatarsal head and one on the fifth metatarsal head).

4 $$ {\overset{\rightharpoonup }{v}}_{dist\_ int}=\left({\overset{\rightharpoonup }{v}}_{foot}-{\overset{\rightharpoonup }{v}}_{dist\_ cable}\right)-{\overset{\rightharpoonup }{\omega}}_{foot}\times \left({\overset{\rightharpoonup }{\rho}}_{foot}-{\overset{\rightharpoonup }{\rho}}_{dist\_ cable}\right) $$

5 $$ {P}_{dist\_ int}=\left(-{\overset{\rightharpoonup }{F}}_{cable}\right)\cdot {\overset{\rightharpoonup }{v}}_{dist\_ int} $$

Again we adopted a convention in which interface power absorption was negative. ${\overset{\rightharpoonup }{F}}_{cable}$ signifies the force pulling downward on the proximal interface, while - ${\overset{\rightharpoonup }{F}}_{cable}$ signifies the force pulling upward on the distal interface.

### Augmentation power

An indirect estimate of augmentation power, *P*_*aug*_*indirect*_, can be calculated by adding each interface power to cable end-effector power. The sum of proximal and distal interface power are simply termed interface power, and represented by *P*_*int*_.

6 $$ {P}_{int} = {P}_{prox\_ int} + {P}_{dist\_ int} $$

7 $$ {P}_{aug\_ indirect} = {P}_{cable\_ end} + {P}_{int} $$

Augmentation power can also be computed more directly using an alternative calculation, which is described in Additional file 1: Figure S3 and shown to yield a power curve very similar to Eqn. 7. However, this direct augmentation power estimate is based on slightly different assumptions than our interface power analysis above. Indirect augmentation power was therefore used in order to maintain mathematical consistency with interface power estimates (per Fig. 2).

### Net ankle power

**Fig. 3:**
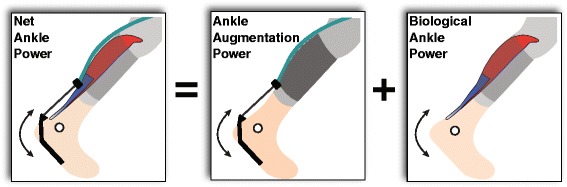
Conceptual summary of ankle power. Net ankle power results from the combination of ankle augmentation power (from the exosuit) and biological ankle power (from muscles, tendons, ligaments). Black arrows represent motions associated with each power term

Net ankle power, *P*_*ankle*_, was computed in 3D via standard inverse dynamics, as the dot product of ankle moment, ${\overset{\rightharpoonup }{M}}_{ankle}$, and ankle angular velocity, ${\overset{\rightharpoonup }{\omega}}_{ankle}$. *P*_*ankle*_ reflects the combined contributions from biological tissues and exosuit augmentation (Fig. 3).

8 $$ {P}_{ankle}={\overset{\rightharpoonup }{M}}_{ankle}\cdot {\overset{\rightharpoonup }{\omega}}_{ankle} $$

### Biological ankle power

Biological contributions to joint power can be estimated indirectly by subtracting ankle augmentation power, *P*_*aug*_*indirect*_, from net ankle power, *P*_*ankle*_. Biological ankle power, *P*_*ankle*_*bio*_*indirect*_, reflects net contributions from muscles, tendons and other tissues acting about the ankle joint.

9 $$ {P}_{ankle\_ bio\_ indirect} = {P}_{ankle} - {P}_{aug\_ indirect} $$

### Idealized augmentation power

For comparison and discussion purposes it is valuable to define one additional power analysis method, idealized analysis, which assumes zero interface losses, and therefore that all of the cable end-effector power contributes directly to augmenting ankle plantarflexion. This yields the following idealized expressions for augmentation power, *P*_*aug*_*ideal*_, interface power, *P*_*int*_*ideal*_, and biological ankle power, *P*_*ankle*_*bio*_*ideal*_:

10 $$ {P}_{aug\_ ideal} = {P}_{cable\_ end} $$

11 $$ {P}_{int\_ ideal} = 0 $$

12 $$ {P}_{ankle\_ bio\_ ideal} = {P}_{ankle} - {P}_{aug\_ ideal} $$

### Summary measures

As summary metrics, we computed positive, negative and net work (mean ± s.d.) for 5 strides during the last 30 s of the trial. Work values were computed over: (i) the *exosuit loading* phase (period of increasing exosuit force application, after pre-tensioning) to capture interface energy absorption dynamics, (ii) the *exosuit unloading* phase to capture interface energy return dynamics, and also (iii) over the full stride cycle. Work values were additionally computed over the pre-tensioning phase (initial period of increasing exosuit force application that removes slack from the exosuit); however, these values were small compared to the other periods (Table 1), and thus not emphasized in results or discussion.

**Table 1:** Net work values (in Joules) are shown for each period of the stride cycle, when peak exosuit forces were 500﻿ N

	Pre-tensioning	Exosuit loading	Exosuit unloading	Full stride
Cable end-effector	0.003 ± 0.1	10.6 ± 0.8	1.6 ± 0.2	12.2 ± 0.8
Proximal interface	−0.8 ± 0.1	−3.9 ± 0.3	3.6 ± 0.3	−1.1 ± 0.1
Distal interface	−0.04 ± 0.02	−2.1 ± 0.3	0.3 ± 0.1	−1.8 ± 0.2
Ankle augmentation	−0.9 ± 0.1	4.7 ± 0.4	5.4 ± 0.5	9.2 ± 0.6

## Results

**Fig. 4:**
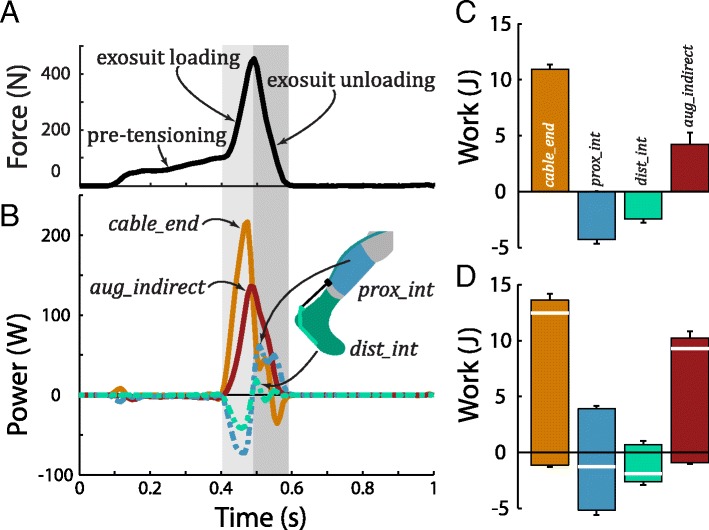
Force, power and work during walking. **a** Exosuit cable force measured via load cell. **b** Cable end-effector power, *P*_*cable*_*end*_ is parsed into power that goes into motion/deformation of the proximal, *P*_*prox*_*int*_, and distal, *P*_*dist*_*int*_, interfaces vs. power that contributes to augmenting ankle plantarflexion, *P*_*aug*_*indirect*_. Power and force results are shown for a representative stride cycle. The left-hand gray box in the background indicates exosuit loading, the primary period of increasing force application. The right-hand gray box indicates exosuit unloading. **c** Positive and negative work (mean ± s.d.) during exosuit loading. **d** Positive and negative work (mean ± s.d.) over the full stride cycle. Net work is indicated by thick white line on each bar

Proximal and distal interfaces each underwent a cycle of negative followed by positive power, resulting in a lower magnitude and later timing of augmentation power relative to cable end-effector power (Fig. 4). These dynamics are summarized quantitatively in the mechanical work metrics below.

### End-effector, interface and augmentation work

Over the full stride cycle, cable end-effector work was 12.2 ± 0.8 J, ankle augmentation work (from *P*_*aug*_*indirect*_) was 9.2 ± 0.6 J, proximal interface work was −1.1 ± 0.1 J, and distal interface work was −1.8 ± 0.2 J (all net work values, Table 1). A breakdown of positive and negative work values is depicted in Fig. 4. Idealized ankle augmentation work was 12.2 ± 0.8 J (i.e., identical to cable end-effector work), since idealized interface work was assumed to be zero.

During exosuit loading, substantial amounts of power were absorbed into the interfaces (Fig. 4, Table 1). The cable end-effector performed 10.6 ± 0.8 J of net work during exosuit loading. Simultaneously, the proximal interface performed −3.9 ± 0.3 J of net work, the distal interface performed −2.1 ± 0.3 J, and 4.7 ± 0.4 J contributed to ankle augmentation. Idealized ankle augmentation work was 10.6 ± 0.8 J.

During exosuit unloading, much of the absorbed interface power was returned viscoelastically, appearing as positive interface work. The proximal interface performed 3.6 ± 0.3 J and the distal interface performed 0.3 ± 0.1 J of net work. Concurrently, the cable end-effector performed some additional net positive work, 1.6 ± 0.2 J. Consequently, 5.4 ± 0.5 J of net work augmented ankle plantarflexion during exosuit unloading. Idealized ankle augmentation work was 1.6 ± 0.2 J.

### Net and biological ankle power

**Fig. 5:**
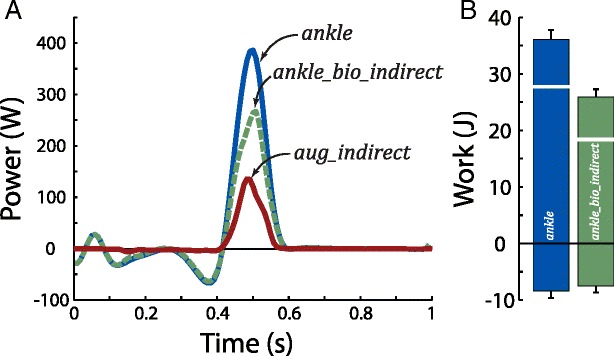
Net ankle power during walking. **a** Net ankle power, *P*_*ankle*_, due to both biological and exosuit contributions, was estimated using standard 3D inverse dynamics. Ankle augmentation power, *P*_*aug*_*indirect*_, represents power provided by the exosuit that augments ankle plantarflexion. Biological ankle power, *P*_*ankle*_*bio*_*indirect*_, reflects the net power generated by muscles, tendons and other biological tissues about the ankle joint. Power results are shown for a representative stride cycle. **b** Positive and negative work (mean ± s.d.) during the walking cycle. Net work is indicated by thick white line on each bar

Over the full stride cycle, 27.5 ± 2.1 J of net ankle work was performed (Fig. 5), ankle augmentation work was 9.2 ± 0.6 J and biological ankle work was 18.2 ± 1.9 J. In contrast, idealized ankle augmentation work was 12.2 ± 0.8 J and idealized biological ankle work was 15.3 ± 2.0 J (15% lower than work from *P*_*ankle*_*bio*_*indirect*_).

During exosuit loading, 15.0 ± 1.7 J of net ankle work was performed, ankle augmentation work was 4.7 ± 0.4 J and biological ankle work was 10.2 ± 1.4 J. In contrast, idealized ankle augmentation work was 10.6 ± 0.8 J and idealized biological ankle work was 4.3 ± 1.5 J (60% lower than work from *P*_*ankle*_*bio*_*indirect*_).

During exosuit unloading, 19.7 ± 1.0 J of net ankle work was performed, ankle augmentation work was 5.4 ± 0.5 J and biological ankle work was 14.3 ± 0.6 J. In contrast, idealized ankle augmentation work was 1.6 ± 0.2 J and idealized biological ankle work was 18.1 ± 0.9 J (25% higher than work from *P*_*ankle*_*bio*_*indirect*_).

## Discussion

There is mounting evidence that wearable assistive devices that generate power, such as exosuits and exoskeletons, can benefit users during walking, [5, 6, 22], hopping [23–25], load carriage [4, 15] and other locomotor tasks, and that these benefits can be realized for individuals across a range of ages [26] and physical abilities [1–3]. But these benefits are only realized when augmentation power is timed and scaled (in magnitude) appropriately; and this augmentation power is heavily dependent on how power is transmitted from a device to the body via physical interfaces. In this study we quantified exosuit-to-human power transmission using new methods that isolate contributions from each human-device interface (proximal and distal). We then presented empirical evidence of how these interfaces absorb substantial power during exosuit loading followed by viscoelastic energy return during unloading, which has important implications for how wearable devices are controlled and coupled to the human body to augment movement. We found that interface dynamics complicate the transmission of power from wearable assistive devices to the human body, resulting in three key consequences: (i) During exosuit loading (as applied forces increased), about 55% of exosuit end-effector power was absorbed into the interfaces. (ii) However, during subsequent exosuit unloading (as applied forces decreased) most of the absorbed interface power was returned viscoelastically. Consequently, the majority (about 75%) of exosuit end-effector work over each stride contributed to augmenting ankle plantarflexion. (iii) Ankle augmentation power (and work) was delayed relative to exosuit end-effector power, due to these interface energy absorption and return dynamics.

### Interface energy absorption and return dynamics

Physical human-device interfaces absorb and return substantial energy (Fig. 4, Table 1). Foremost, our findings highlight the pitfall of neglecting interface power absorption (as with the idealized analysis), which masks the reshaping of augmentation power due to energy absorption/return (Fig. 4), and the resulting changes to biological joint power (Fig. 5). Furthermore, our results reveal the means by which energy storage and return mechanisms enable the majority of cable end-effector work (~75%) to contribute to ankle augmentation. The primary energy storage and return mechanism was due to stretching/recoiling of the interface materials and biological tissues (the second mechanism, due to cable recoil, is discussed below). During the exosuit loading phase, about 55% of the cable end-effector work was absorbed into the interfaces (roughly 35% proximal, and 20% distal, Fig. 4, Table 1), while about 45% augmented ankle plantarflexion (given peak force of 500 N). Next, during exosuit unloading, the interfaces returned the majority of this absorbed power, and this contributed to augmenting ankle plantarflexion (positive interface power and work in Fig. 4, Table 1). We also observed evidence of a secondary, smaller energy storage and return mechanism, due to stretching/recoiling of the inner Bowden cable (between the backpack-mounted actuator unit and the end-effector). Evidence of this cable recoil power is visible in Fig. 4 as the extra positive peak in *P*_*cable*_*end*_ in the middle of exosuit unloading. As a result of these interface and cable recoil dynamics, the majority of ankle augmentation work was actually performed during the exosuit unloading phase (as applied forces decreased), and not during exosuit loading (as forces increased) as one might intuit. This then caused a time lag in peak augmentation power relative to cable end-effector power (Fig. 4). Of note, the energy storage and return cycle as well as the delayed peak augmentation power observed in this study were qualitatively consistent with prior observations on exosuit-assisted walking [11].

In the future, device (internal) power transmission analysis could also be performed to provide a more comprehensive estimate of Bowden cable dynamics. These estimates could be combined with the device-to-human analysis presented here to yield a more complete mapping of power generation and losses during transmission from the actuator to the human user. Internal device power analysis requires an additional load sensor at the actuator unit itself, which was not available in this study (e.g., [11]).

### Implications on physical interface design

Exosuit-to-human power transmission may be improved (in terms of timing and/or magnitude) by advances in physical interfaces. Improved power transmission would enable lighter batteries and actuators (while maintaining performance), or enable higher levels of performance (with the same device hardware). In this study we quantified the behavior of a single shank (proximal) and single boot (distal) interface. However, in future studies multiple “candidate” interfaces could be compared to identify the best interface (e.g., in terms of minimizing interface power loss or augmentation power time lag). Physical interfaces could potentially benefit from a number of design refinements. Stiffer interface materials may reduce power absorption during exosuit loading due to textile stretching, whereas more highly damped materials may reduce energy return during unloading (if minimizing recoil dynamics was desirable for an application). Physical interfaces could also be designed to distribute loads over larger skin area or to load only targeted areas of the body, in order to reduce power due to deformation of the skin and underlying biological tissues. Furthermore, physical interfaces could be designed with materials that better adhere to and grip the human body, to reduce power due to motion of the interface relative to the skin.

### Implications on device control

Control algorithms could also be optimized to reduce interface losses and/or compensate for the observed time lag in augmentation power by incorporating models of physical interface dynamics. New controllers might, for instance, be designed to use predictive algorithms in conjunction with new sensor suites to begin actuation earlier (i.e., preemptively) in order to ensure that augmentation power is provided at the most beneficial instant or phase of movement. Timing of augmentation power is known to be important for walking [27, 28], and we anticipate that it will be even more important for tasks with higher rates of loading and power, such as running, jumping, cutting or landing. For these tasks, incorrect or imprecise timing of augmentation power could render the device ineffective, detrimental or potentially even dangerous to the user; thus highlighting the need for controllers that account for interface dynamics.

Future control algorithms may also be able to facilitate improved device-to-human power transmission by exploiting force-dependent interface dynamics. During the first minute of the walking trial, peak cable forces slowly ramped up. In these data, we observed that when peak forces of 250 N were applied, the proximal and distal interfaces collectively absorbed about 80% of the cable end-effector work during exosuit loading and returned the majority of this power during unloading (Additional file 1: Table S1). However, when peak forces were doubled to 500 N, the percentage of energy absorbed during exosuit loading decreased to 55% (Table 1), indicative of stiffening interface behaviors. This suggests that increasing actuator forces may not only increase the magnitude of exosuit power delivered but also the fraction of this power that contributes directly to augmentation during exosuit loading; potentially representing dual benefits to providing higher forces, so long as forces can be applied safely and comfortably.

### Benefits of new method for quantifying interface dynamics

In this study we presented a new methodology capable of quantifying power contributions from human-device interfaces during dynamic movement. Our method offers two key benefits relative to previously published approaches. First, this new method isolates power contributions from each individual interface, whereas previous methods either provide a lumped interface power estimate (see Additional file 1) or completely neglect interface dynamics (*P*_*int*_*ideal*_). In this study we assessed a monoarticular (ankle only) exosuit, but the benefits of quantifying individual interface contributions are most evident when one considers evaluation of more complex, multiarticular devices [29]. For instance, a bilateral lower-body exosuit could have 7 or more interfaces (one interface for the pelvis, and one for the foot, shank, and thigh of each leg). Previous augmentation power analysis methods provide a lumped estimate of power from all 7 interfaces, whereas the interface power analysis presented here could be applied to provide an interface-by-interface breakdown (i.e., 7 separate interface power curves). Quantifying separate contributions from each interface would be helpful in identifying weak links in the design and improving sub-optimal interfaces or device components. Second, interface power analysis circumvents the need to conduct a series of time-consuming, component-by-component pre-experiments (to estimate stiffness and damping parameters which are then used to model interface contributions during movement). Rather this new approach enables us to directly estimate all interface powers simultaneously during movement tasks, using common motion capture and force measurements. Interface power analysis can be performed together with standard inverse dynamics analysis (Fig. 5), which makes it practical and inexpensive to incorporate into existing or planned experimental evaluations. Finally, we note that the methods summarized here are intended and expected to be generalizable to other wearable assistive devices (e.g., rigid exoskeletons), which is important for evaluating and comparing devices in the future, given that interface and augmentation dynamics are expected to be highly task- and device-specific.

### Limitations and future work

This study has a few notable limitations. First, in this initial investigation, only one soft exosuit prototype, one joint and one subject were evaluated. This was sufficient to achieve our objective of presenting new methods that can isolate interface power absorption, and which could be easily extended to assess other wearable assistive devices, including both rigid exoskeletons and soft exosuits. The case study data presented provide proof-of-concept of the analysis approach, and useful benchmark data that highlights how interface dynamics can complicate power transmission from wearable assistive devices to the human body. Future studies are warranted to investigate inter-subject variability (e.g., due to age, weight or body morphology), additional tasks, additional body joints and segments, and various devices and physical interface designs. Second, this newly proposed analysis isolates individual interface dynamics, but in theory each interface power estimate could be further decomposed into contributions from the device interface components (e.g., textiles) vs. biological tissues vs. relative motion between the device and skin; though experimentally this decomposition may be challenging without additional pre-experiments [17] or measurement modalities. Third, small inaccuracies in estimates are expected due to limitations of skin-mounted markers [30]. Markers were placed to minimize motion due to skin/tissue deformation, and small displacements due to skin motion were not expected to affect general interpretations. Fourth, this study focuses on the effects of interface power absorption, but several other outcome measures (not quantified here) are also of interest and importance when evaluating human-exosuit interface performance. These include interface migration over time (i.e., displacement of the device interface relative to the skin due to slippage, which can affect device performance), compression and shear pressures provided by the interface (which can affect skin health and integrity), and perceived user comfort (which would likely affect device usage). Each of these factors can improve or degrade the performance benefits received by the user, and thus these additional outcomes provide complementary information to help guide the design of wearable assistive devices. Incorporating these measures into future wearable assistive device studies will provide a more comprehensive understanding of human-device interaction dynamics.

## Conclusion

Physical interfaces, although often neglected, can absorb and return substantial energy and thereby complicate power transmission. Here we present a new methodology that can isolate power contributions from individual human-device interfaces. This provides insight into device-to-human power transmission and how to improve the design and control of wearable assistive devices. In order to optimize the performance of wearable assistive devices and fully realize their potential human augmentation benefits it is important, throughout design and evaluation phases, to anticipate and account for human-device interface dynamics that affect power transmission.

## Additional file

Additional file 1: Supplementary methods and results, including additional details on the motion capture marker set, calculations of cable end-effector, augmentation and interface powers, a comparison of the direct vs. indirect power estimates, and work values estimated while walking with lower peak exosuit forces of 250 N. (PDF 515 kb)

## Abbreviations

*P*_*aug*_*ideal*_: idealized ankle augmentation power; equivalent to cable end-effector power *P*_*cable*_*end*_
${\overset{\rightharpoonup }{\omega}}_{ankle}$: absolute angular velocity of the ankle
${\overset{\rightharpoonup }{\omega}}_{foot}$: absolute angular velocity of the foot segment
${\overset{\rightharpoonup }{\omega}}_{shank}$: absolute angular velocity of the shank segment
${\overset{\rightharpoonup }{\rho}}_{dist\_ cable}$: absolute position of the distal cable marker in the lab coordinate frame
${\overset{\rightharpoonup }{\rho}}_{foot}$: absolute position of the foot in the lab coordinate frame
${\overset{\rightharpoonup }{\rho}}_{prox\_ cable}$: absolute position of the proximal cable marker in the lab coordinate frame
${\overset{\rightharpoonup }{\rho}}_{shank}$: absolute position of the shank in the lab coordinate frame
${\overset{\rightharpoonup }{v}}_{dist\_ cable}$: absolute translational velocity of the distal cable marker
${\overset{\rightharpoonup }{v}}_{foot}$: absolute translational velocity of the foot segment
${\overset{\rightharpoonup }{v}}_{prox\_ cable}$: absolute translational velocity of the proximal cable marker
${\overset{\rightharpoonup }{v}}_{shank}$: absolute translational velocity of the shank segment
${\overset{\rightharpoonup }{r}}_{cable\_ end}$: cable end-effector length vector
${\overset{\rightharpoonup }{F}}_{cable}$: cable force vector
*P*_*aug*_: direct estimate of ankle auentation power
*P*_*dist*_*int*_: distal interface power
${\overset{\rightharpoonup }{v}}_{dist\_ int}$: distal interface velocity; velocity of the distal interface force application point relative to the foot’s gross (rigid-body) motion
*P*_*ankle*_*bio*_*ideal*_: idealized biological ankle power; computed as the difference between net ankle power *P*_*ankle*_, and idealized augmentation power, *P*_*aug*_*ideal*_
*P*_*int*_*ideal*_: idealized interface power; always zero because idealized method assumes no interface dynamics
*P*_*aug*_*indirect*_: indirect estimate of ankle augmentation power; the difference between cable end-effector power *P*_*cable*_*end*_, and total interface power, *P*_*int*_
*P*_*ankle*_*bio*_*indirect*_: indirect estimate of biological ankle power; in this study computed as the difference between net ankle power *P*_*ankle*_, and augmentation power, *P*_*aug*_*indirect*_
*P*_*int*_*indirect*_: indirect estimate of total interface power; the difference between cable end-effector power *P*_*cable*_*end*_, and augmentation ankle power, *P*_*aug*_
${\overset{\rightharpoonup }{r}}_m$: moment arm of the cable acting about the ankle joint center
${\overset{\rightharpoonup }{M}}_{ankle}$: net ankle moment
*P*_*ankle*_: net ankle power; calculated via standard 3D inverse dynamics
*P*_*cable*_*end*_: power generated at the exosuit cable end-effector
*P*_*prox*_*int*_: proximal interface power
${\overset{\rightharpoonup }{v}}_{prox\_ int}$: proximal interface velocity; velocity of the proximal interface force application point relative to the shank’s gross (rigid-body) motion
*P*_*int*_: total interface power; the sum of proximal and distal interface powers
${\overset{\rightharpoonup }{u}}_{cable\_ end}$: unit vector oriented along the cable at the end-effector, defined from the distal to the proximal cable marker
${\overset{\rightharpoonup }{v}}_{cable\_ end}$: velocity of the cable at the end-effector Some abbreviations are defined in Additional file 1.
