# Supplementary material for: Physical interface dynamics alter how robotic exosuits augment human movement: implications for optimizing wearable assistive devices
Source: J Neuroeng Rehabil. 2017 May 18;14:40. doi: 10.1186/s12984-017-0247-9 (PMC5437613; doi:10.1186/s12984-017-0247-9)
Supplement: Additional file 1: — Supplementary methods and results, including additional details on the motion capture marker set, calculations of cable end-effector, augmentation and interface powers, a comparison of the direct vs. indirect power estimates, and work values estimated while walking with lower peak exosuit forces of 250 N. (PDF 515 kb) [file 12984_2017_247_MOESM1_ESM.pdf]

Appendix

Supplementary methods and results are provided below, including additional details on the motion capture marker set, calculations of cable end-effector, augmentation and interface powers, a comparison of the direct vs. indirect power estimates, and work values estimated while walking with lower peak exosuit forces of 250 N.

Supplementary Methods

Motion Capture Marker Set

The full motion capture marker set is provided in Fig. S1, to expand upon the simplified representation in Fig. 1 of the main text.

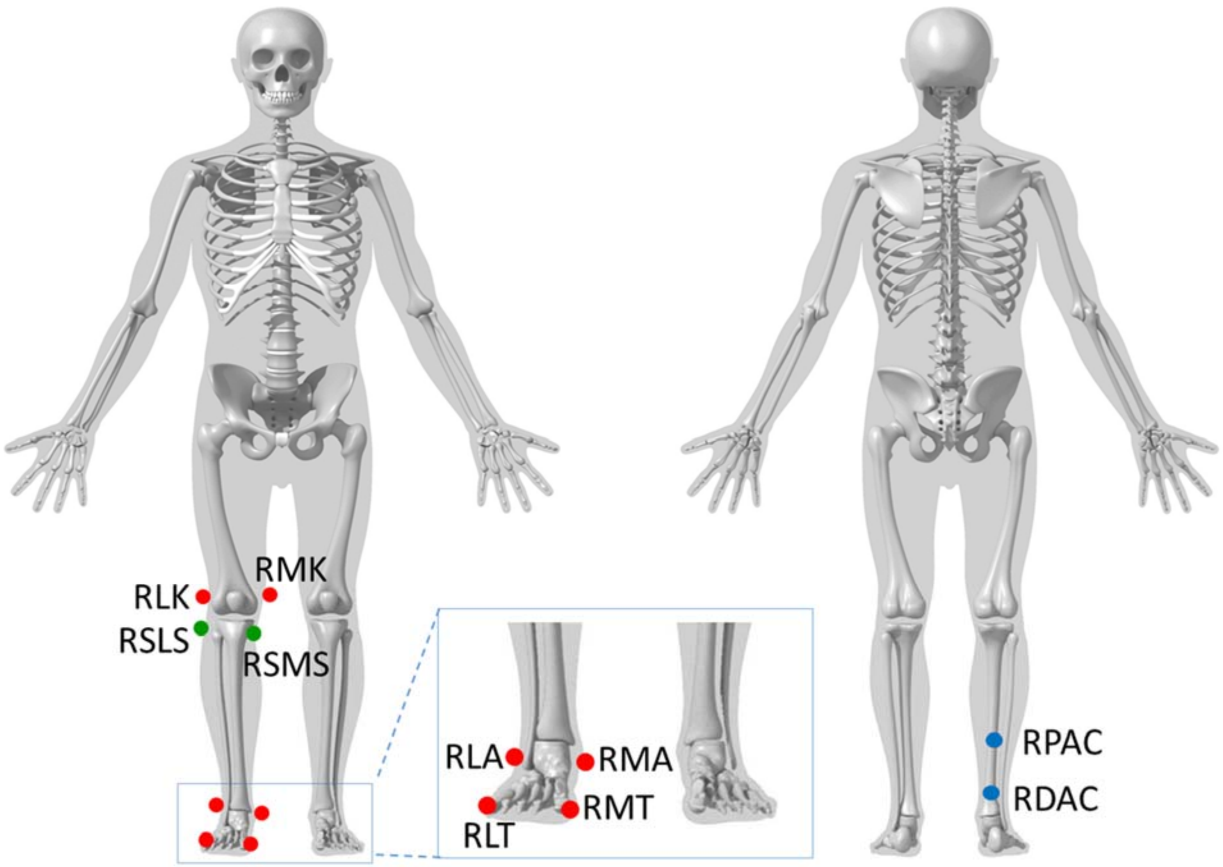

Figure S1. Full marker set. Anterior (left): RLK: Right Lateral Femoral Epicondyle; RMK: Right Medial Femoral Epicondyle; RLA: Right Lateral Malleolus; RMA: Right Medial Malleolus; RLT: Right 5th Metatarsal Head; RMT: Right 1st Metatarsal Head; RSLS: Right Superior Lateral Shank; RSMS: Right Superior Medial Shank. Posterior (right): RPAC: Right Proximal Ankle Cable; RDAC: Right Distal Ankle Cable. Shank kinematics were estimated using RLK, RMK, RLA, RMA, RSLS, and RSMS. Foot kinematics were estimated using RLA, RMA, RMT, and RLT. Cable end-effector kinematics were estimated using RPAC and RDAC.

Cable End-Effector Power Derivation

We start by defining the cable end-effector length vector, $\vec{r}_{cable\_end}$, and the unit vector, $\vec{u}_{cable\_end}$, oriented along the cable at the end-effector by measuring the position of the proximal cable marker, $\vec{\rho}_{prox\_cable}$, relative to the distal cable marker, $\vec{\rho}_{dist\_cable}$ (Fig. 1).

$$\vec{r}_{cable\_end} = \vec{\rho}_{prox\_cable} - \vec{\rho}_{dist\_cable} \tag{S1}$$

$$\vec{u}_{cable\_end} = \frac{\vec{r}_{cable\_end}}{|\vec{r}_{cable\_end}|} \tag{S2}$$

The length estimate defined by $\vec{r}_{cable\_end}$ assumes that the cable end-effector is taut (straight-line connection), which is true in this exosuit when non-negligible forces are applied. The measured load cell force magnitude borne by the cable, F_{cable}, is directed along the line of action of the cable end-effector, yielding the force vector in 3D space, $\vec{F}_{cable}$, which we defined as oriented in the opposite direction of $\vec{u}_{cable\_end}$.

$$\vec{F}_{cable} = F_{cable} \cdot (-\vec{u}_{cable\_end}) \tag{S3}$$

Velocity of cable lengthening/shortening along the line of action of the cable end-effector, $\vec{v}_{cable\_end}$, can then be computed as:

$$\vec{v}_{cable\_end} = \frac{d}{dt}(\vec{r}_{cable\_end} \cdot \vec{u}_{cable\_end}) \cdot \vec{u}_{cable\_end} \tag{S4}$$

With this convention, increasing negative values of $\vec{v}_{cable\_end}$ signify increasing shortening velocity of the cable.

Finally, we can compute power due to length changes of the cable end-effector, P_{cable_end}, by computing the dot product of cable force and velocity vectors, yielding Eqn. 1 in the main text.

Direct Augmentation Power and Indirect Interface Power

In the main text, ankle augmentation power, $P_{aug\_indirect}$, was estimated using what we term an indirect approach (adding interface power to cable end-effector power). An alternative way to compute augmentation power, which we term the direct method and denote as P_{aug}, is by taking the dot product of the applied force and the velocity due to joint rotation [5], [11], [22], [31]–[33]. In this study, this velocity was calculated by taking the cross product of the ankle angular velocity, $\vec{\omega}_{ankle}$, based on a rigid-body link-segment model, and the moment arm, $\vec{r}_m$, of the cable acting about the estimated ankle joint center. The moment arm was calculated as the perpendicular distance between the ankle joint center and the line of cable action.

$$P_{aug} = \vec{F}_{cable} \cdot (\vec{\omega}_{ankle} \times \vec{r}_m) \tag{S5}$$

Subtracting cable end-effector power, P_{cable_end}, from augmentation power, P_{aug}, then provides an indirect estimate of total interface power. We maintained the convention from the main text in which interface power absorption was negative. We refer to this estimate as *indirect interface power*, $P_{int\_indirect}$, because it represents what is left over after accounting for augmentation power contributions. This indirect estimate cannot localize or partition power contributions due to individual interfaces (e.g., proximal vs. distal).

$$P_{int\_indirect} = P_{aug} - P_{cable\_end} \tag{S6}$$

A conceptual comparison of analysis methods is shown in Fig. S2: (A) starting from direct augmentation power estimates, then indirectly estimating total interface power, (B) starting from direct interface power estimates, then indirectly estimating augmentation power, or (C) using idealized analysis in which interface dynamics are assumed to be zero (non-existent).

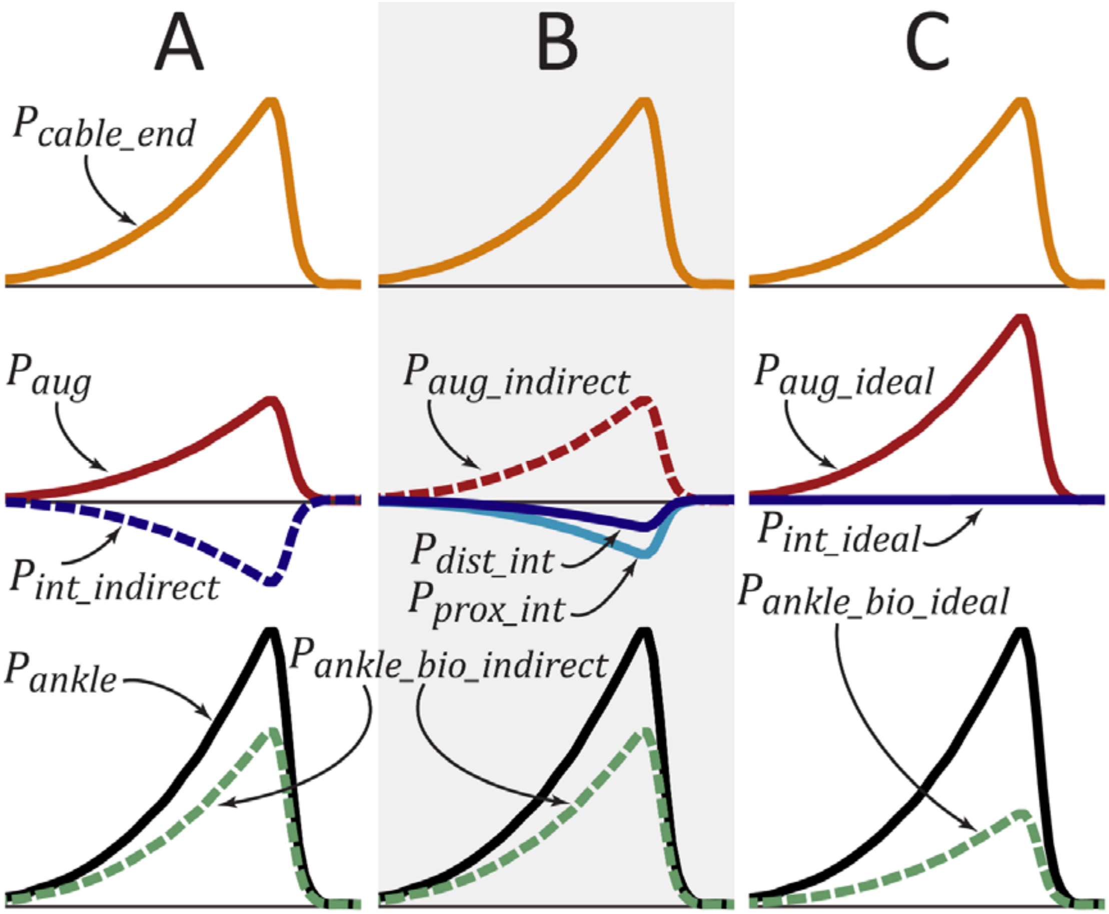

Figure S2. Conceptual summary of three analysis methods for estimating device-to-human power transmission. Each column (A-C) depicts one power analysis method. The top row represents the end-effector power (P_{cable_end}, orange line, i.e., power output from the device at the cable end-effector, assumed to be the same for each method), the middle row partitions this power into augmentation (red) vs. interface power estimates (blue), then the bottom row depicts the resultant biological power contributions. (A) Augmentation power (P_{aug}, solid red) can be computed directly, then total interface power ($P_{int\_indirect}$, dashed dark blue) can be calculated indirectly by subtracting P_{cable_end} from P_{aug}. (B) Proximal (P_{prox_int}, solid light blue) and distal (P_{dist_int}, solid dark blue) interface powers can be computed directly, then augmentation power ($P_{aug\_indirect}$, dashed red) can be calculated indirectly by adding the summed interface powers, P_{int}, to P_{cable_end}. (C) Using idealized analysis, interface power (P_{int_ideal}) is assumed to be zero, and therefore P_{aug_ideal} is equal to P_{cable_end}. For each method, biological ankle power (dashed green) can be estimated indirectly by subtracting augmentation power from inverse dynamics ankle power (P_{ankle}, black). Methods (A) and (B) are expected to yield similar biological power estimates, $P_{ankle\_bio\_indirect}$. The benefit of method (B) is that individual interface contributions can be partitioned. Idealized analysis, as shown in column (C), is expected to greatly overestimate augmentation power and underestimate biological power ($P_{ankle\_bio\_ideal}$), due to neglected interface dynamics. Indirect power estimates (i.e., computed by adding/subtracting power terms) are shown as dashed lines. Direct power estimates (i.e., computed by multiplying force by velocity, or torque by angular velocity) are shown as solid lines. No power units are shown because this is a conceptual/explanatory representation, not data.

Supplementary Results

Direct vs. Indirect Power Estimates

We found that direct and indirect estimates yielded similar interface power and similar augmentation power curves (Fig. S3). Both interface power estimates (P_{int} and $P_{int\_indirect}$) followed a similar pattern of energy absorption and return. Ankle augmentation power was positive for both methods (P_{aug} and $P_{aug\_indirect}$), with similar peak magnitudes and timing. In terms of work, direct vs. indirect augmentation work during exosuit loading phase was 4.7 ± 0.4 J vs. 4.7 ± 0.4 J, and during exosuit unloading 6.6 ± 0.7 J vs. 5.4 ± 0.5 J, respectively. Minor quantitative differences are to be expected due to measurement limitations and assumptions inherent in each estimation method. However, since these calculations are based on different underlying assumptions, the strong agreement between curves (Fig. S3) gives confidence in the general magnitudes and waveforms of augmentation and interface powers.

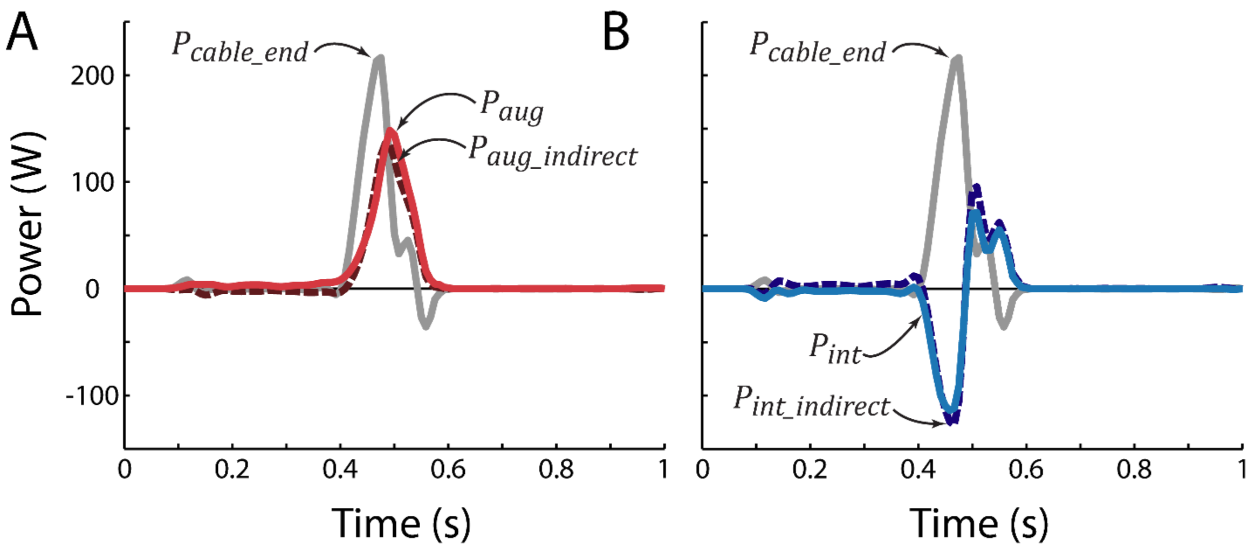

Figure S3. Direct vs. indirect power estimates. Direct and indirect estimates yielded similar curves for augmentation power, and also for interface power. (A) Augmentation power: direct (P_{aug}, solid red) vs. indirect ($P_{aug\_indirect}$, dashed red) estimate. (B) Interface power: direct (P_{int}, solid blue) vs. indirect ($P_{int\_indirect}$, dashed blue) estimate. A single representative stride cycle is depicted. For reference, P_{cable_end} (light gray) is also shown.

Work Values at Lower Peak Exosuit Force

Work values are provided in Table S1 based on data from the first minute of the walking trial, during which exosuit forces gradually ramped up.

Table S1. Net work values (in Joules) are shown for each phase of the stride cycle, when peak exosuit forces were 250 N.

	Pre-Tensioning	Exosuit Loading	Exosuit Unloading	Full Stride
Cable End-Effector	-0.1 ± 0.03	3.4 ± 0.5	0.6 ± 0.2	3.8 ± 0.7
Proximal Interface	-0.4 ± 0.04	-1.8 ± 0.2	1.7 ± 0.2	-0.5 ± 0.1
Distal Interface	0.1 ± 0.02	-1.0 ± 0.2	0.1 ± 0.04	-0.8 ± 0.2
Ankle Augmentation	-0.5 ± 0.1	0.6 ± 0.1	2.3 ± 0.4	2.4 ± 0.4
